# Retraction: Research on risk identification of manufacturing enterprises’ Internet strategic transformation

**DOI:** 10.1371/journal.pone.0332092

**Published:** 2025-09-11

**Authors:** The PLOS One Editors

The *PLOS One* Editors retract this article [1] because it was identified as one of a series of submissions for which we have concerns about potential manipulation of the publication process, peer review integrity, and authorship. These concerns call into question the validity and provenance of the reported results. We regret that the issues were not identified prior to the article’s publication.

In addition, the Editors have concerns regarding the reference list, including the addition during revision of multiple references that do not appear to be relevant in the cited context. The corresponding author stated that these references were included to demonstrate methodological rigor and alignment with established research practices.

HH and GHKZ did not agree with the retraction. HA either did not respond directly or could not be reached.
